# Clinical outcomes in neonates following maternal magnesium sulfate therapy in preeclampsia/eclampsia

**DOI:** 10.1186/cc10926

**Published:** 2012-03-20

**Authors:** G Tikhova, E Shifman

**Affiliations:** 1 Kulakov Scientific Center of Obstetrics, Gynecology and Perinatology, Moscow, Russia

## Introduction

Magnesium sulfate therapy (MST) is the method of choice in prophylaxis and treatment of eclamptic seizures in many countries. A lot of high-quality clinical trials and meta-analyses proved its efficacy and safety for mothers. But the effect of maternal MST on the fetus and neonate is still controversial. The goal of the study was to analyze available trials concerning this problem in order to prove statistically that maternal MST given as prophylaxis or treatment of eclamptic seizures has no adverse effects on the mature fetus and term neonate.

## Methods

Trials were searched for in the PubMed database among English-language articles published in 1990 to 2010. Analysis includes randomized controlled prospective clinical trials comparing MST with no treatment, placebo or other anticonvulsant. The following neonatal outcomes were chosen as the main endpoints of the study: neonatal death, neonatal hypotonia, Apgar score <7 at 1 and 5 minutes, intubation at place of delivery, admission to the NICU, treatment in NICU >7 days. The total effect of MST was measured as the relative risk of adverse outcome in the MST group compared with control and its 95% CI. Meta-analysis of neonatal outcomes was performed under a random-effect model for seven endpoints and a fixed-effect model for three endpoints.

## Results

Neonatal mortality in the MST group was compared with different control groups. Each of these studies showed no significant difference between two groups: MST/mixed (0.89, 95% CI 0.80 to 0.99), MTS/no treatment-placebo (0.99, 95% CI 0.93 to 1.05), MTS/diazepam (1.09, 95% CI 0.91 to 1.29), MTS/fenitoin (0.75, 95% CI 0.56 to 1.02). The neonatal hypotonia rate is significantly higher in the MST group (3.57, 95% CI 2.89 to 4.42), although significant heterogeneity of the control group may be a valuable confounding factor. There was no evidence for changing incidence of Apgar <7 at 1 and 5 minutes in the MTS group compared with control (0.79, 95% CI 0.70 to 0.89 and 0.80, 95% CI 0.64 to 0.99 correspondingly). The same results were observed for intubation at place of delivery (1.04, 95% CI 0.90 to 1.29) and admission to NICU (0.96, 95% CI 0.85 to 1.08). The incidence of treatment in the NICU >7 day was significantly lower in MST group than in control (0.54, 95% CI 0.52 to 0.78).

## Conclusion

Maternal MST given as prophylaxis or treatment of eclamptic seizures does not affect neonatal mortality and incidence of neonatal hypotonia, Apgar <7 at 1 and 5 minutes, intubation at place of delivery and admission to the NICU in a population of term newborns. Maternal MST significantly reduces the risk of neonate treatment in NICU >7 days in this population.

